# Circulating Cytokines Predict ^1^H-Proton MRS Cerebral Metabolites in Healthy Older Adults

**DOI:** 10.3389/fnagi.2021.690923

**Published:** 2021-08-19

**Authors:** Kailey Langer, Ronald A. Cohen, Eric C. Porges, John B. Williamson, Adam J. Woods

**Affiliations:** ^1^Center for Cognitive Aging and Memory, Clinical Translational Research Program, College of Medicine, University of Florida, Gainesville, FL, United States; ^2^Department of Clinical and Health Psychology, College of Public Health and Health Professions, University of Florida, Gainesville, FL, United States

**Keywords:** aging, MRS, cerebral metabolites, cytokines, tumor necrosis factor-α

## Abstract

**Background**: Changes in both circulating cytokines and neurochemical concentrations have been observed in aging. Patterns of change across these factors are associated with age-related pathologies, including neurodegenerative disease. More evidence to define patterns of change that are characteristic of healthy aging is needed, as is an investigation into how age-related changes in blood cytokines and brain neurochemicals may relate to one another in a healthy older adult population.

**Methods**: Single voxel ^1^H-proton magnetic resonance spectroscopy was collected in medial frontal and medial parietal regions. Phosphocholine and glycerophosphocholine (Cho), *myo-*inositol (MI), N-acetylaspertate and N-acetylasperglutamate (NAA), creatine and phosphocreatine (Cr), and glutamate and glutamine (Glx) were measured in a sample of 83 healthy, cognitively normal adults aged 52–89. Blood data were collected to quantify 12 cytokines: interleukins (IL-) 2, 5, 6, 7, 8, 10, 12, 13, IL-1 β, tumor necrosis factor α (TNF-α), interferon γ (IFN-γ), and IL-17 α. Correlation analyses were performed to assess age relationships between each of these factors. Backward linear regressions were performed. Cytokine data and age were used as predictors of each cerebrospinal fluid (CSF)-corrected metabolite concentration in both voxels.

**Results**: Associations were identified between a variety of cytokines and concentrations of frontal NAA, Cr, and Glx, and of parietal MI, Cho, NAA, and Cr. In the frontal voxel, NAA was predicted by more IL-1B and less TNF-α, Cr by less TNF-α and more IL-5, and Glx by less TNF-α. In the parietal voxel, MI was predicted by more IL-10 and IL-8 and less IL-2, Cho by more TNF-α and less IL-2, NAA by more IL-1B and TNF-α and less IL-13, IL-2, and IL-7, and Cr by more IL-10 and less IL-2.

**Conclusions**: Associations were identified between circulating cytokines and neurometabolite concentrations in this sample of older adults. The present results serve as the initial evidence of relationships between circulating cytokines and neurophysiology. Findings invite further investigation to understand the physiological consequences of aging, and how peripheral inflammatory markers may relate to neurochemical concentrations in healthy aging.

## Introduction

There is well-documented evidence of age-related changes in a variety of physiological processes that impact health and functional outcomes. Several biological theories of aging may contextualize these observations. The free radical theory of aging (Harman, 1956), for example, attributes aging to cellular damage incurred from the accumulation of reactive oxygen species (ROS). This acquired oxidative stress damage may then contribute to chronic low-grade inflammation. An alternative theory focuses on the role of genomic instability in driving the biological changes observed in aging (López-Otín et al., 2013). Some additionally propose an evolutionary perspective, suggesting that aging is a deliberate and adaptive aspect of life (Goldsmith, 2012). More recent approaches have taken an integrative perspective, though more research is needed to understand mechanisms of change throughout the lifespan. Changes in peripheral inflammatory agents that occur with advancing age may impact structural, functional, and neurochemical outcomes in the aging brain. The present study focuses on the role of inflammatory changes, investigating peripheral inflammatory markers and neurochemical concentrations in the context of non-pathological aging.

Cytokines are immune signaling molecules that can be measured through blood. Cytokines modulate inflammatory responses, and circulating cytokines are useful inflammatory markers for study. Specific cytokines have more often been investigated in the context of aging, in particular interleukin 6 (IL-6; Ershler, 1993; Forsey et al., 2003; Miwa et al., 2016). Tumor necrosis factor α (TNF-α) also has been investigated frequently in the context of both healthy and pathological aging. Studies have shown elevated circulating TNF-α in aging (Paolisso et al., 1998; Bruunsgaard et al., 1999). Recent evidence has demonstrated relationships between TNF-α and volumetric brain measures, as well as a measure of global cognitive performance in functionally intact older adults (Lindbergh et al., 2020). Evidence has shown that TNF-α is associated with a variety of age-associated morbidities, including atherosclerosis (Bruunsgaard et al., 2000), dementia (Bruunsgaard et al., 1999), type 2 diabetes (Borst, 2004), and sarcopenia (Bian et al., 2017), as well as mortality (Bruunsgaard et al., 2003; Roubenoff et al., 2003).

Many other pro-inflammatory cytokines have additionally been identified, along with evidence to support their roles in a variety of age-related pathologies. However, there is less consistent evidence to suggest reliable patterns of change across these cytokines in healthy older adult populations (Minciullo et al., 2016; Rea et al., 2018). Some studies have suggested potential implications for longevity related to increased anti-inflammatory cytokine concentrations (Franceschi et al., 2007). This may suggest that for some individuals co-occurring increases in anti-inflammatory activity, alongside increases in pro-inflammatory activity, serve in a compensatory role.

Increases in pro-inflammatory activity, and indications of oxidative stress damage, also have been observed in the aging brain and in neurodegenerative disease. Although the mechanisms of oxidative damage in the brain are not thoroughly defined, neuroinflammatory signaling has been described as one byproduct of ROS released from neuronal metabolism (Castelli et al., 2019). Low-grade, chronic neuroinflammation is characterized by the proliferation of microglia and astrocytes (two primary cell types involved in the cerebral immune response), as well as increases in IL-6, TNF-α, and interleukin 1 β (IL-1β) in the central nervous system (Yin et al., 2016). Proton magnetic resonance spectroscopy (MRS) is a technique that allows for non-invasive *in vivo* quantification of metabolites in the brain (Ross and Bluml, 2001). From the perspective of increased pro-inflammatory activity in aging, certain patterns of change may be expected across these markers. Prior studies have identified decreases in N-acetylaspartate (NAA; a commonly used marker of neuronal density; Marjańska et al., 2017), and increases in *myo*-inositol (MI; a commonly used marker of neuroinflammation; Raininko and Mattsson, 2010) in older compared to younger adults. While there is a body of evidence supporting these trends (Cichocka and Bereś, 2018), MRS literature has been somewhat mixed regarding age-related changes across commonly measured cerebral metabolites (Cleeland et al., 2019). This may be due to methodological differences in MRS studies which may not allow for comparable interpretations of results across studies. Further, there has been some debate about the precise roles of metabolites. For example, some have linked both mI and choline (Cho) to neuroinflammatory processes (Chang et al., 2013); others have suggested alternative roles of Cho and that the role of mI is more nuanced than that of a glial marker (Rae, 2014).

These metabolites may serve as markers of energy expenditure, neuronal density, or neuroinflammation. Assessment of neurochemical concentrations in aging or neurodegenerative disease may be sensitive to earlier changes than those captured by structural brain imaging. If neurochemical alterations are assumed to be a precursor to further age-related volumetric changes, it stands to reason that these alterations would follow a similar anatomical progression and occur more dramatically in areas known to be vulnerable to aging. Decades of literature have established that regional patterns of atrophy are observed in addition to the whole-brain volumetric loss in normal aging (Scahill et al., 2003; Raz et al., 2005). Findings indicate that frontal brain tissue is particularly susceptible to age-related changes. The present study used this framework to investigate neurochemical concentrations in a frontal voxel and a parietal voxel, under the assumption that these areas may diverge in terms of neurochemical concentrations observed in aging.

It is unclear whether inflammatory processes occur in tandem in central and peripheral nervous systems. It is important to investigate whether there is a relationship between peripheral inflammatory markers and cerebral metabolites. Each has been observed to change throughout healthy aging, and relate to age-related pathologies such as dementia. Cerebral metabolites have been investigated in the context of other populations with elevated pro-inflammatory profiles, including individuals with HIV (Harezlak et al., 2014). Several studies additionally have explored the relationship between circulating cytokines and neurochemical concentrations in the context of specific inflammation-related illnesses (e.g., neonatal encephalopathy, acute liver failure, hepatitis C), but no prior studies were identified that investigated relationships in a healthy older adult sample (Bartha et al., 2004; Gupta et al., 2010; Haroon et al., 2014; Taylor et al., 2014).

The present study explores age relationships with circulating cytokines and cerebral metabolites to identify whether previously observed age relationships exist in this sample. Greater pro-inflammatory cytokines (e.g., IL-6, TNF-α) and cerebral metabolites measured by ^1^H-proton MRS are expected to be associated with older age. The primary aim is to investigate whether circulating cytokines predict neurometabolites in a sample of healthy older adults. It is hypothesized that an increased presence of pro-inflammatory cytokines will be associated with increased cerebral metabolites implicated in neuroinflammation (MI, Cho), and decreased markers of brain health or function (NAA, Cr, Glx). The opposite is hypothesized for the greater presence of anti-inflammatory cytokines.

## Materials and Methods

### Participants

Eighty-three older adults (range 52–89; 61% female) were recruited from the local community as volunteers for a study on healthy aging (i.e., without clinical symptoms of mild cognitive impairment or dementia, or subjective cognitive complaint). Participants were cognitively assessed as part of the study procedure; summary statistics for these tests and participant demographics are described in Table 1. Unadjusted Montreal Cognitive Assessment (MoCA) scores ranged between 20 and 30. There has been some debate in the literature regarding optimal MoCA cutoff scores for distinguishing normal cognition from mild cognitive impairment. In addition to this screening tool, a preponderance of neuropsychological evidence available for each participant was used to determine cognitive status, and ensure the sample excluded those showing clinical symptoms of impairment. Mean scores reported for all neuropsychological measures, excluding MoCA, reflect t-scores calculated from an age-matched normative population. Individuals with a self-reported history of neurological or psychiatric brain disorders, a diagnosis of a neurodegenerative disease, or MRI ineligibility were excluded from the study. Informed consent was obtained from all participants and all study procedures were approved by the University of Florida Institutional Review Board.

**Table 1:** Demographics and cognitive profile.

Demographics	Mean (SD)
Age	72.46 (8.98)
Education	16.73 (2.48)
Sex (F/M)	51/32
Charlson Comorbidity Index	0.82 (1.68)
**Neuropsychological Test Scores**	
MoCA	26.02 (2.55)
Immediate Recall	55.23 (11.11)
Speed/Executive Function	49.79 (9.79)
Confrontation Naming	56.73 (10.47)
Verbal Fluency	48.19 (11.63)

*Education refers to number of years; MoCA, Montreal Cognitive Assessment education-adjusted total raw score; Immediate Recall = California Verbal Learning Test-II (CVLT-II) trials 1–4 normative t-score; Speed/Executive Function = Trail Making Test Part B normative t-score; Confrontation Naming = Boston Naming Test (BNT) normative t-score; Verbal Fluency = F-A-S verbal fluency normative t-score*.

### MRS Acquisition and Processing

**Figure 1:**
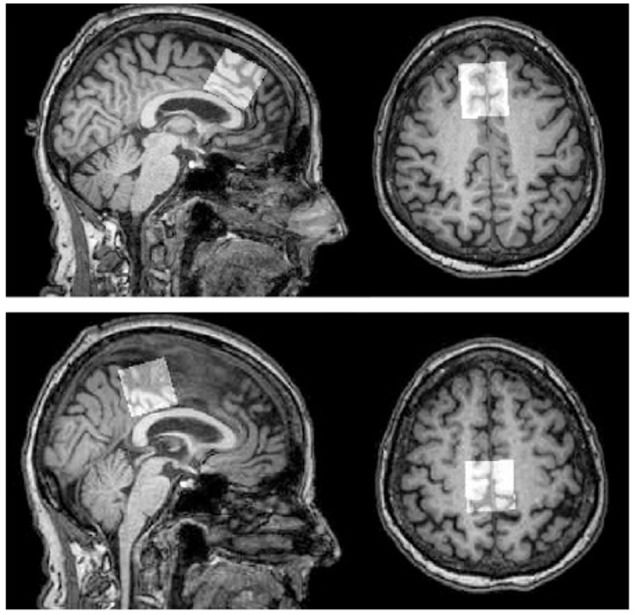
MRS voxel placement. Top panel: frontal voxel placement, superior to the genu of the corpus callosum, aligned with corpus callosum, medial on an axial plane. Bottom panel: parietal voxel placement, posterior and superior to the corpus callosum, aligned with the corpus callosum, medial on an axial plane. *Note*: Figure published elsewhere (Porges et al., 2017).

Scanning was performed on a 3T Philips Achieva scanner (Philips Healthcare, Best, The Netherlands), using a 32-channel head coil. Single-voxel ^1^H spectra PRESS MRS were collected in two voxels, in the anterior and posterior regions (for placement see Figure 1). A T1-weighted anatomical image (magnetization-prepared rapid gradient-echo; repetition time/echo time 5 8 ms/3.7 ms, 1-mm^3^ isotropic voxels) was acquired for MRS voxel placement and segmentation. GannetCoRegister software (Harris et al., 2015) was used in data collection. Tissue fractions were derived, and an expert rater confirmed appropriate voxel placement based on landmarks in individual subject anatomical space. Parameters were optimized for primary analyses using MEGA-PRESS data collection. Each voxel was 3 × 3 × 3 cm (27 cm^3^). The PRESS sequence had a repetition time of 2 s and an echo time of 68 ms. Acquisition variables included: 320 transients with editing ON-OFF scans alternating every two transients, a 16-step phase cycle with steps repeated for on and off, 2048 data points acquired at a spectral width of 2 kHz, and variable pulse power and optimized relaxation delays (VAPOR) water suppression at a width of 80 Hz. No fat suppression was used. Editing-OFF spectra were acquired for use in the present dataset, and research has been done to validate this method (Dhamala et al., 2019). Sixteen transients of water-unsuppressed data were also acquired for quantification using the same acquisition variables. Osprey (Oeltzschner et al., 2020) was run for pre-processing, and LC Model spectral analysis software (Provencher, 2001) with the water-unsuppressed acquisition was used to determine absolute values, in mmol per Kg wet weight (mM), of the following metabolites: NAA (N-Acetylaspartate plus N-Acetylaspartylglutamate), Cr (creatine plus phosphocreatine), MI (*myo*-inositol), Glx (glutamate plus glutamine), and Cho (glycerophosphocholine plus phosphocholine). Water scaling and eddy-current correction settings were set to true; other LCModel settings were kept as default (ppmst = 4.0, ppmend = 0.2). The MRS metabolites measured, along with their proposed roles, are described in Table 2. Metabolite values were excluded based on a corresponding percent standard deviation of 20 or greater (calculated in LC Model) which has been used as a rough criterion for determining acceptable reliability (Provencher, 2021). This resulted in different sample sizes across the different metabolite concentrations obtained.

**Table 2:** Descriptions of MRS metabolites measured in this study.

MRS metabolite	Abbreviation	Physiological/metabolic function in the brain
Glycerophosphocholine + Phosphocholine	Cho	Choline-containing compounds, essential for maintaining the structural integrity and signaling of cell membranes. Increased Cho concentrations are indicative of greater membrane turnover in the brain, and often are elevated in response to neuroinflammation.
Creatine + Phosphocreatine	Cr	Creatine is a compound synthesized by the liver and kidneys and is essential for metabolism in muscle and the brain. Cr enables the recycling of ATP by facilitating the conversion of ADP back to ATP. Cr provides vital energy stores for the brain and other tissues.
*myo*-Inositol	MI	A carbocyclic sugar in the brain and other tissues, mediates cell signal transduction in response to neurotransmitters, hormones, and growth factors. MI also influences osmoregulation, and in the brain is responsive to glial activation, with increased concentrations often indicative of neuroinflammation.
N-Acetylaspartate + N-Acetylaspartylglutamate	NAA	NAA is an amino acid that is present in high concentrations in the brain, synthesized and stored primarily in neurons. NAA contributes to energy production from glutamate neuronal mitochondria, and in the brain is located in neuronal cell bodies and fibers, and thus serves as a neuronal marker that is indicative of neuronal integrity. NAA is also an osmolyte involved in fluid balance in the brain, and is a source for lipid and myelin synthesis.
Glutamate + Glutamine	Glx	Glutamate is an essential excitatory neurotransmitter, active throughout the brain. Glutamate is often reduced in the context of brain disease, indicating reduced excitatory neurotransmitter capacity, though very elevated glutamate levels can also be neurotoxic. Glutamine is an amino acid with many physiological functions, including in digestion.

*Table reproduced from Cohen et al. (in press)*.

### Cytokine Measurement

Once blood samples were acquired, plasma from each sample was separated, aliquoted, and immediately stored at −80°C. Aliquots were used to measure cytokine levels using an xMAP multiplexed bead array. Using the Luminex-100 system (Luminex Corp., Austin, TX, USA), multiple cytokines were simultaneously quantified by capturing them onto antibody-coated, spectrally distinct fluorescent microspheres and measuring fluorescence intensity. In some cases, cytokine measurements fell below a detectable value, and in those cases, the threshold for detectability was used. The cytokines measured in picograms permilliliter (pg/ml), along with their proposed functions, are described in Table 3.

**Table 3:** Descriptions of cytokines measured in this study.

Cytokine	Abbreviation	Function
**Pro-Inflammatory**		
Interferon gamma-soluble cytokine	IFN-γ	Produced by innate NK cells, acquired antigen-specific cytotoxic CD4+ and effector CD8+ T cells. Activates macrophages and critical for innate and adaptive immune responses to intracellular pathogens, tumor control, and inhibition of viral replication.
Tumor necrosis factor-α	TNF-α	Secreted by macrophages, monocytes, neutrophils, T cells, NK cells after stimulation with LPS. CD4+ cells secrete TNF-α. Also made by astrocytes, microglial cells, smooth muscle cells, and fibroblasts. Mediates systemic inflammation, inhibits viral replication, and inhibits tumorigenesis.
Interleukin-1 Beta	IL-1 β	Produced by activated macrophages; mediates inflammatory responses, cell proliferation, apoptosis. Induces Cox-2 in CNS, causing inflammatory pain.
Interleukin-6	IL-6	Secreted by T cells and macrophages; triggers inflammation, acute phase response, fever. Anti-inflammatory effects include inhibiting TNF-α and IL-1 and activating IL-1ra and IL-10.
Interleukin-8	IL-8	Made by macrophages and some epithelial and endothelial cells; Role in the innate immune response. Major role in chemotaxis of neutrophils. Also mediates inflammatory response and angiogenesis.
Interleukin-17-Alpha	IL-17 α	Produced by a subset of CD4 T cells. Can stimulate the expression of IL-6. Pro-inflammatory cytokine needed to eliminate extracellular bacteria and fungi. Associated with chronic inflammatory diseases including rheumatoid arthritis, psoriasis, and multiple sclerosis.
**Anti-Inflammatory**		
Interleukin-10	IL-10	Produced by monocytes. Pleiotropic cytokine. As an anti-inflammatory cytokine, it inhibits macrophage and dendritic cell function, suppresses TNF-α. Acquires pro-inflammatory activity during immune response with IFN-g stimulation.
Interleukin-12	IL-12	Produced mostly by phagocytic cells. Stimulates production of IFN-g from T and NK cells. Involved in Th1 and Th2 cell differentiation and proliferation, and optimal IFN-g production in response to antigens.
**Adaptive Immunity**		
Interleukin-2	IL-2	Produced primarily by CD4+ T cells. T cell growth factor, important for the proliferation of T and B lymphocytes.
Interleukin-5	IL-5	Produced by Th2 cells and mast cells. Acts as a growth and differentiation factor for B cells and eosinophils. Involved in the regulation of eosinophil formation, maturation, recruitment, and survival.
Interleukin-7	IL-7	Important for B and T cell development, essential components of the immune system. Produced locally by intestinal epithelial and epithelial goblet cells. May play a role in lymphoid cell survival.
Interleukin-13	IL-13	Immunoregulatory cytokine produced primarily by activated Th2 cells. Involved in B-cell maturation and differentiation. Inhibits production of pro-inflammatory cytokines and chemokines.

*Table recreated in part from Cohen et al. (2011) and Turner et al. (2014). Although cytokines are broadly categorized as pro- or anti-inflammatory, notably many cytokines serve to regulate immune responses and can therefore serve either role*.

### Statistical Analysis

IBM SPSS Statistics version 25 (IBM Corp., Armonk, NY, USA) was used to perform all analyses. Absolute values of metabolites were corrected for cerebrospinal fluid (CSF) by computing [Metabolite/(1-CSF)] for each. This was performed to address the potential contribution of individual differences in tissue fraction within voxels to metabolite quantities. Tissue correction is particularly relevant in an older adult population where age-related cortical atrophy may drive differences in uncorrected metabolite values. Normality was assessed, and cytokine data were log-transformed to assess skewness. All variables included in the models were standardized by z-scoring to facilitate interpretation of the results. Simple bivariate Pearson correlations were performed to explore the relationships between age and each cytokine and cerebral metabolite in the current sample. Cytokine concentrations and age were entered as predictors of MRS concentrations in the anterior and posterior voxels in backward stepwise regression models. Independent analyses were conducted for each MRS concentration in both voxels. MRS metabolites were entered into each model as the dependent variables; independent variables across all models included circulating cytokines, sex, and age.

## Results

**Figure 2:**
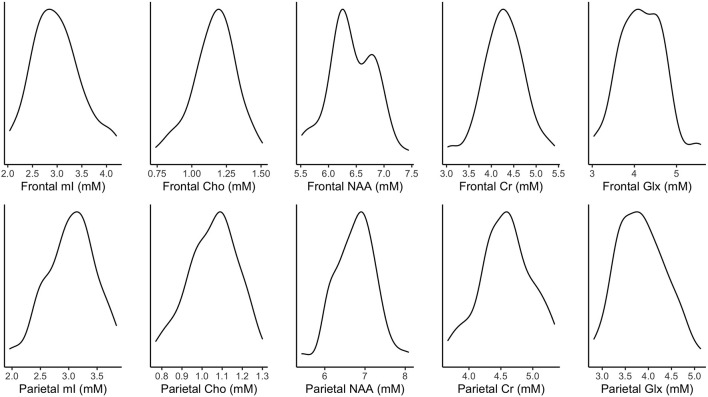
Density plots of tissue-corrected metabolites. mM, mmol per Kg wet weight.

**Figure 3:**
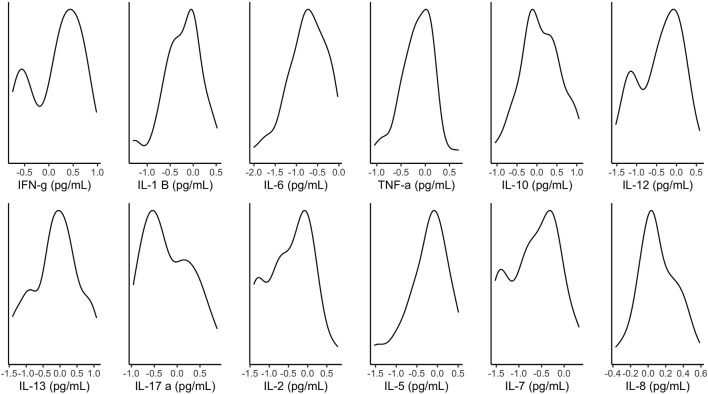
Density plots of log-transformed cytokines. pg/mL, picograms permilliliter.

Figure 2 illustrates the distribution of metabolites corrected for tissue fraction in the present sample. Figure 3 illustrates the distribution of log-transformed cytokines.

### Relationships Between Age, Circulating Cytokines and MRS Metabolites

These values represent the original data prior to the statistical transformations described above. Findings in Table 4 demonstrate that, within this sample of older adults, there was nosubstantial evidence of age relationships across the majority of included neurometabolites and cytokines. The strongest age relationships identified were between frontal NAA concentrations and TNF-α, such that greater age was related to decreased NAA measured in the frontal voxel, and increased circulating TNF-α. Notably, *p*-values for correlations presented here were not corrected for multiple comparisons, and when corrected no significant age associations survived.

**Table 4:** Correlation results, cytokines and tissue-corrected metabolites related to age.

Voxel	Metabolite	Correlation	Cytokine	Correlation
Anterior	MI	0.082	IFN-γ	−0.039
	Cho	−0.094	IL-1 β	0.001
	NAA	−0.251*	IL-6	0.058
	Cr	−0.009	TNF-α	0.238*
	Glx	0.060	IL-10	0.024
			IL-12	0.030
Posterior	MI	0.041	IL-13	−0.041
	Cho	0.097	IL-17 α	−0.055
	NAA	−0.023	IL-2	0.087
	Cr	−0.013	IL-5	−0.047
	Glx	−0.085	IL-7	−0.015
			IL-8	0.109

Results from the larger matrix of correlations are shown for relationships between age and each metabolite and cytokine measured; *p < 0.05.

### Cytokines Predicting MRS Metabolites

Table 5 shows the results of the backward linear regressions run for anterior voxel data, using cytokine data to predict concentrations of five metabolites. Age did not survive the final model for any metabolites in the frontal voxel. TNF-α was a significant negative predictor for NAA, Cr, and Glx concentrations. IL-1 β was a significant predictor of NAA. This effect was opposite to the hypothesized direction, such that greater circulating IL-1 β was associated with greater frontal NAA. Likewise, pro-inflammatory cytokine IL-5 was a positive predictor of Cr in the frontal voxel. There were no cytokines that survived the final models for MI and Cho.

**Table 5:** Regression results, cytokines predicting frontal MRS metabolites.

Metabolite	Cytokine (Predictor)	*b* (se)	*p*-value	*R* ^2^	*n*
NAA	IL-1 β	0.318 (0.140)	0.026	0.112*	78
	TNF-α	−0.422 (0.138)	0.003		
Cr	TNF-α	−0.265 (0.118)	0.028	0.083*	78
	IL-5	0.251 (0.119)	0.038		
Glx	TNF-α	−0.279 (0.134)	0.041	0.067*	62

b, unstandardized b-weight; *se*, standard error; *n*, sample size; *p < 0.05.

Table 6 shows the results of the backward linear regressions run for posterior voxel data, using cytokine data to predict concentrations of five metabolites. Variations in sample size were due to the listwise exclusion of missing values. Age did not survive the final model for any metabolites in the parietal voxel. Findings suggest IL-2 is an important predictor for parietal neurochemical concentrations. IL-2 negatively predicted parietal MI, Cho, NAA, and Cr. As a pro-inflammatory cytokine, its negative direction was expected in models predicting NAA and Cr, but not in those predicting MI and Cho. MI concentrations also were positively predicted by IL-10 and IL-8. The positive direction of IL-10 in this model also was unexpected, particularly because IL-10 was a positive predictor of Cr as well. TNF-α positively predicted both Cho and NAA. NAA also was positively predicted by IL-1 β and was negatively predicted by IL-13 and IL-7. The directions of the relationships of IL-1 β and TNF-α in this model were inconsistent with our hypotheses. There were no cytokines that survived the final model for Glx.

**Table 6:** Regression results, cytokines predicting parietal MRS metabolites.

Metabolite	Cytokine (Predictor)	*b* (se)	*p*-value	*R* ^2^	*n*
MI	IL-10	0.329 (0.158)	0.041	0.183**	74
	IL-2	−0.494 (0.153)	0.002		
	IL-8	0.231 (0.112)	0.043		
Cho	TNF-α	0.409 (0.157)	0.011	0.139**	76
	IL-2	−0.517 (0.151)	0.001		
NAA	IL-1 β	0.580 (0.237)	0.017	0.173*	76
	TNF-α	0.312 (0.167)	0.067		
	IL-13	−0.281 (0.124)	0.027		
	IL-2	−0.579 (0.254)	0.026		
	IL-7	−0.310 (0.179)	0.088		
Cr	IL-10	0.380 (0.159)	0.019	0.126*	76
	IL-2	−0.503 (0.155)	0.002		

b, unstandardized b-weight; *se*, standard error; *n*, sample size; *p < 0.05; **p < 0.005.

## Discussion

Present findings contribute to the body of literature describing age-associated changes across both cytokines and cerebral metabolites. The broad hypothesis that associations exist between cytokines and cerebral metabolites was supported by the evidence presented. However, data from this sample failed to provide consistent evidence in support of hypotheses about directionality. Also of interest are the unexpected parallel effects of cytokines across different and in some cases functionally oppositional, cerebral metabolites concentrations. The differences observed in effects across frontal and parietal metabolites are noteworthy as well. Changes in both peripheral inflammation and neurophysiology have been observed both in pathological (i.e., neurodegenerative disease), and healthy aging (Yin et al., 2016; Rea et al., 2018). The organizing perspective guiding the present study design was that inflammatory changes are central to systemic, age-related physiological changes. That perspective is more closely in line with the free radical theory of aging. However, the lack of consistency in the directionality of the relationships may serve as support for a more integrated view of the biological mechanisms of aging. To provide context for evaluating the relationships identified between circulating cytokines and cerebral metabolites in the present sample, it is relevant to assess whether relationships between these variables and age are present as context for the primary analyses.

### Age, Cytokines, and MRS Metabolites Relationships

The literature is inconsistent regarding expected cerebral metabolite changes in cognitively normal older adults. This inconsistency, though, may be attributable to methodological variability. Findings are not always comparable for a number of reasons, including differences in the procedure for tissue correction, acquisition parameters, and processing and reporting data (Cleeland et al., 2019). Null findings in the present study across the majority of cerebral metabolites contribute to the growing body of literature investigating age-related neurochemical changes. Though no *p*-values survived correction for multiple comparisons, a larger sample size may result in more pronounced effects. The effects identified by uncorrected criteria are described below. It is possible that age-associated changes are not as clearly detectable within a group of cognitively normal, healthy older adults, though the present sample had a fairly broad age range. NAA has long been regarded as a neuronal marker due to early studies using tumors and cultured cells to demonstrate detectable NAA in neurons but not in other cell types (Nadler and Cooper, 1972; Urenjak et al., 1992). The present data suggest a relationship between frontal NAA and age, such that decreased NAA was related to increased age, which is consistent with prior studies evidencing age-associated decreases in NAA (Brooks et al., 2001; Ding et al., 2016). As a marker of neuronal density, NAA may be associated with measures of structural brain volume. Frontal decreases in NAA parallel established patterns of frontal lobe cortical volume reductions in older adults (Resnick et al., 2003; Pfefferbaum et al., 2013).

Although prior literature supports an association between age and IL-6 (Ershler, 1993; Forsey et al., 2003), data from the present sample were not able to detect that association. One potential explanation may have to do with participants’ medical health relative to those included in prior studies, though objective medical health data were not collected as part of the larger trial and therefore were not available for this analysis. Ridker et al. (2000) found an association between IL-6 and myocardial infarction after controlling for other cardiovascular risk factors, and Ferrucci et al. (2005) showed a much smaller predictive effect of age for IL-6 after cardiovascular factors were taken into account. It is possible that IL-6 has more to do with cardiovascular health than it does with chronological age. While age and cardiovascular health are related, the degree of heterogeneity for health outcomes in aging introduces some implicit variance. As previously stated, this analysis utilized data from a study deliberately targeting non-pathological aging.

An association between increased age and increased TNF-α was found in the present sample. This is consistent with prior literature demonstrating increases in TNF-α production in healthy older, compared with younger, adults (Fagiolo et al., 1993; Zanni et al., 2003). TNF-α has been an important cytokine in prior literature investigating age-related inflammation. TNF-α is a multifunctional, pro-inflammatory cytokine implicated in a variety of disease states, as well as in chronic systemic inflammation. Findings have demonstrated relationships between TNF-α and cognitive impairment and dementia (Bruunsgaard et al., 1999), incidence of cardiovascular events (Cesari et al., 2003), insulin resistance (Borst, 2004), frailty (Michaud et al., 2013), and mortality. A 10-year study by Varadhan et al. (2014) found that TNF-α predicted all-cause mortality in a sample of older adults, and Roubenoff et al. (2003) additionally demonstrated the relationship between TNF-α and mortality in a population-based sample of older adults over a 4-year period (Roubenoff et al., 2003; Varadhan et al., 2014).

### Relationships Between Cytokines and MRS Metabolites

The hypotheses for the present analyses were based on the suppositions: (a) that inflammatory changes occur in healthy aging and drive the development of age-associated morbidities, including pathological brain changes; (b) that peripheral and cerebral inflammatory processes may be parallel such that greater pro-inflammatory cytokines circulating peripherally would correspond with greater metabolic indications of cerebral neuroinflammatory processes; and (c) that greater anti-inflammatory cytokines circulating may relate to some compensatory processes that would correspond with greater metabolic indications of brain health. Evidence has supported that increases in circulating pro-inflammatory cytokines are related to worse outcomes for cardiovascular health and neurodegenerative disease (Bruunsgaard et al., 1999; Cesari et al., 2003; Miwa et al., 2016). It was predicted that more of a classically pro-inflammatory marker would negatively predict a marker of neuronal health and density. However, in some cases, the opposite trend was found.

TNF-α appeared to have a particularly important predictive value across metabolites in both voxels. With specific regard to its negative effects on frontal NAA, Cr, and Glx concentrations, the significance of TNF-α is interesting considering the relative strength of its relationship with age. It is noteworthy that findings indicate TNF-α, above and beyond chronological age, was predictive of neurochemical changes that may occur in pathological aging (Olson et al., 2008; Zeydan et al., 2017). One potential interpretation is that these effects are indicative of greater inflammation, explaining variance in neurochemical changes that may otherwise be attributed to aging. Brain changes also occur in preclinical phases of neurodegenerative diseases, before clinical symptoms may be identified. Further, TNF-α has been associated with diseases (e.g., artherosclerosis, type 2 diabetes, sarcopenia) often related to pathological aging, as well as dementia and all-cause mortality (Bruunsgaard et al., 1999, 2000, 2003; Borst, 2004; Bian et al., 2017). Perhaps the observed relationships between increased TNF-α and decreased frontal NAA and Glx specifically represent a risk of pathological aging outcomes in this sample, and chronological age is less relevant. Of course, in a cross-sectional analysis, it is not possible to say for certain whether that may be the case.

The differential effects observed for cytokines are of potential interest. For example, TNF-α negatively predicted frontal NAA but positively predicted NAA in the parietal voxel. Prior literature has established a differential impact of aging on structural brain outcomes (Raz et al., 2005). As neurochemical changes may serve as a precursor to atrophy, it would follow that certain areas are more vulnerable to age-related metabolic changes than others. An investigation of the relationships between cerebral metabolites and both cortical and subcortical brain volumes in the same sample has recently been published and found divergent relationships between frontal and parietal metabolite concentrations and cortical and subcortical brain volumes (Williamson et al., 2021). Additionally, in many cases, cytokines are pleiotropic or act on each other which further complicates the interpretation of seemingly counterintuitive effects. IL-10, for example, has been regarded as anti-inflammatory and considered in the context of promoting longevity (Lio et al., 2002), but also has some pro-inflammatory roles (e.g., enhancing B cell survival and proliferation). In this sample, IL-10 positively predicted a marker of neuroinflammation, and also positively predicted a marker of energy production. Further, research particularly related to mood disorders have identified a positive relationship between inflammation and elevated extracellular glutamate (Haroon and Miller, 2017; Haroon et al., 2017). Present findings identified a negative relationship between TNF-a and Glx; however, models of glutamate excitotoxicity are important to consider in the context of this study which did not control for participants’ mood.

There is not yet the depth of knowledge provided by additional studies on the topic to comment on the mechanism by which they each are associated. Findings suggest inter-related degrees of circulating cytokines and neurochemical concentrations that may not appear straightforward at baseline in a healthy aging sample, but that may have implications for future progression of various health outcomes. The current sample was clinically healthy at the time of assessment. However, it is possible that stratifying participants by cardiovascular health factors or future conversion to dementia would show a clearer picture of the way circulating cytokines and cerebral metbaolites interact. That also may help to inform about the transition from cognitively normal, “healthy” older adult to pathological aging. The nature of the present findings may suggest that differences in circulating cytokines better predict changes related to age-associated pathologies. Inclusion of alternative biological mechanisms of change (e.g., mitochondrial dysfunction, hypometabolism) may be important for characterizing brain changes occurring in non-pathological aging.

### Limitations and Future Directions

The present study had several limitations worth considering which may help guide future research endeavors. First, the study design was cross-sectional, preventing any causal interpretation of the results. It is possible to speculate about the differences between previous studies’ methodologies or samples in the context of inconsistent evidence across these markers; however, longitudinal data would be useful in determining both how cytokines and MRS-measured cerebral metabolites may change and relate to one another in healthy aging. One methodological consideration is the use of absolute values rather than ratios. The present study assessed only absolute values; however, the use of ratios in future research may add valuable information about the nature of neurochemical alterations in aging.

Another limitation may have been the use of absolute values of individual cytokines. An important direction for future work, considering the sometimes counterintuitive effects of cytokines found here, is to investigate the presence of non-linear relationships. Modeling relative rather than absolute concentrations of cytokines also may be helpful in fully understanding their collective impact, as many are pleiotropic and regulate the production or proliferation of other cytokines. Further, future incorporation of other related factors would be helpful in understanding the complex relationships between cytokines and cerebral metabolites in older adults. Some factors of interest may be objective cardiovascular and general medical health measures to provide context for inflammatory markers, as well as diffusion tensor measures of white matter integrity and quantification of central nervous system cytokines in CSF samples. It would be valuable to compare levels of peripheral and central circulating cytokines, and to assess whether central circulating cytokines have more direct relationships to cerebral metabolites. Prior research has identified relationships between CSF chemokines and MRS-measured metabolites in an HIV population (Letendre et al., 2011). It also will be important to assess sex differences in the relationships between neurometabolites and circulating cytokines. Sex differences previously have been reported both in circulating cytokines (Klein and Flanagan, 2016) and MRS-measure metabolites (Grachev and Apkarian, 2000).

### Conclusions

The present study introduces relationships between cerebral metabolites and circulating cytokines in a population in which both have implications for the progression of health outcomes, including neurodegenerative disease. The present findings illustrate that these associations are nuanced and complex, but also may serve as preliminary evidence of the interconnectedness of inflammatory markers and neurometabolites in a presently healthy population. This provides a basis for further investigation. It has long been understood that inflammatory markers are important predictors of cognitive decline, dementia, cardiovascular health, and mortality in older adults. Certain pro-inflammatory cytokines have more often been investigated in the context of aging research (e.g., IL-6, TNF-α, IL-1 β). The findings in this sample also provide a basis for allocation of research exploring a broader collection of cytokines in healthy aging, as there are implications of a greater number of inflammatory markers for brain health in this population. Further exploration of this topic is relevant to building more well-informed risk profiles for cognitive decline and dementia, if patterns of circulating cytokines may be useful in predicting neurochemical changes characteristic of preclinical neurodegenerative disease and pathological aging.

## Data Availability Statement

The raw data supporting the conclusions of this article will be made available by the authors, without undue reservation.

## Ethics Statement

The studies involving human participants were reviewed and approved by University of Florida Institutional Review Board. The patients/participants provided their written informed consent to participate in this study.

## Author Contributions

Each author made substantial contributions to the formation of the project, and the execution of the manuscript. All authors contributed to the article and approved the submitted version.

## Conflict of Interest

The authors declare that the research was conducted in the absence of any commercial or financial relationships that could be construed as a potential conflict of interest.

## Publisher’s Note

All claims expressed in this article are solely those of the authors and do not necessarily represent those of their affiliated organizations, or those of the publisher, the editors and the reviewers. Any product that may be evaluated in this article, or claim that may be made by its manufacturer, is not guaranteed or endorsed by the publisher.
